# Adrenarche as a regulator of sensitivity to early adversity

**DOI:** 10.1111/jne.70149

**Published:** 2026-02-24

**Authors:** J. Herbert

**Affiliations:** ^1^ John van Geest Centre for Brain Repair, Department of Clinical Neurosciences University of Cambridge Cambridge UK

**Keywords:** adrenarche, dehydroepiandrosterone, early adversity, glucocorticoids, plasticity

## Abstract

The human brain is highly sensitive to early adversity, which can have long‐term consequences for later mental health. It is also a time of rapid learning of social, motor and other skills, including language. It is proposed that pre‐adrenarche, the only epoch in human development in which cortisol is not accompanied by dehydroepiandrosterone (DHEA) and its sulphated derivative (DHEAS), represents the sensitive period but this is subsequently moderated by the advent of adrenarche and the surge of DHEA(S) at 6–8 years. Cortisol enhances plasticity and the formation of new memories as well as personality traits such as emotionality. DHEA(S) is well known to oppose many of the actions of cortisol on the brain, including those on learning, memory and synaptic function, all reflecting altered plasticity; adrenarche is therefore a time of moderated cortisol activity. Several endocrine‐dependent neural mechanisms respond to the neuroendocrine transition at adrenarche, including alterations in perineuronal nets, gene expression of growth factors, serotonin activity, cytokine release and synaptic adaptability. Adrenarche will reduce the detrimental impact of adverse events but stabilise memories and psychological traits acquired during the cortisol‐dominated pre‐adrenarche epoch. The transition from pre‐ to post‐adrenarche is therefore a highly significant neuroendocrine event in early life, with both potentially beneficial and disadvantageous consequences. This suggests a primary role for adrenarche, for which no function has yet been established.

## 1 THE CRITICAL PERIOD FOR LEARNING

The first few years are notable for rapid learning of skills, of which language is a prime example. This reflects the ability of the young brain to acquire knowledge and adapt to novel experiences.^1^, ^2^ It is much harder to learn a new language, and speak it like a native, after the first few years^3^, ^4^, ^5^ and this is also true for new motor skills (e.g., skiing).^6^, ^7^, ^8^ Specific memories enable rapid learning about these new elements of the world, whether advantageous or potentially damaging. But early events also shape the brain's developing structure, including synaptogenesis, which will play a prominent role in later neural function. This represents a period of heightened sensitivity to incoming information, one example being the visual system (more particularly, the visual cortex) which defines the age range that restricting or depriving the function of one eye has on sight.^9^, ^10^ Critical periods are a more general phenomenon during development and define the age when sensory experience is necessary to establish optimal cortical representations of the surrounding environment.^1^, ^2^ Enhanced sensitivity to early experience has adaptive advantages. Very young infants need to be able to learn quickly, as they progress from total dependence on their parents to increasing independence in a highly complex and challenging world. Regulation of plasticity in later life is necessary to ensure high‐fidelity representations of sensory events and the development of complex skills.^1^

The postnatal brain is also, for the first few years of life, highly sensitive to adversity.^11^, ^12^ Adverse experiences during this time can have long‐term consequences for cognitive and emotional development as well as increasing the risk for later physical and mental disorders.^13^, ^14^, ^15^ Poverty, parental neglect or absence, physical or psychological abuse, poor housing, social or environmental disasters or deficiencies, mental disorder in one or more parent, the horrors of war are some of the roots of early deprivation or adversity, though the precise definition of what constitutes adversity, and how to assess it, are still debated.^16^, ^17^, ^18^, ^19^ The first 6–8 years are also characterised by high levels of plasticity and adaptability in the developing brain.^20^ Adversity influences this maturational and interactive process, which normally results in an orderly progression of developing motor, cognitive, language, emotional, and social skills, influenced by interactions between genetic make‐up and environmental events such as the intricate interplay between mother and child.^21^, ^22^ This is not to say that adversity later in life is without consequences, but responses to such incidents will depend not only on the contemporary events themselves but how brain function has been shaped by experience and adversities and the individual response to them during early life.^14^, ^23^ For example, changes in schooling and the onset of puberty are among challenges that may have considerable influence on the development of the brain and consequent behaviour.^24^, ^25^ Adolescence is also characterised by negative reactions and mental dysfunction to more proximate adverse life events, but these can be influenced by earlier circumstances.^26^ However, adverse events occurring after early childhood have less drastic effects on the brain and are more easily reversible.^1^ One hypothesis accounting for the persistent influence of early adversity is a mismatch between experience of events during the first few post‐partum years and later conditions: another is that early and later life events represent a ‘double‐hit’ that risks adverse psychological, affective and cognitive states.^27^, ^28^ Plasticity during early life is thus a double‐edged sword that facilitates both adaptation and vulnerability.^29^

Experimental and clinical evidence shows that early adversity results in structural changes in the brain,^30^ in changes in synaptic function and pruning and altered connectivity in the amygdala and associated areas of the brain^31^, ^32^, ^33^, ^34^, ^35^ as well as altered epigenetic function, a potentially powerful mechanism for the enduring effects of the early environment^15^, ^36^, ^37^, ^38^, ^39^, ^40^, ^41^ and on individual differences in the expression and variations in neural genes at a time when these are highly active during early post‐natal life.^42^, ^43^ For example, variations in the BDNF and catechol‐O‐methyl transferase genes can moderate the impact of adversity on brain development,^44^, ^45^ but polygenic risk scores give a more complete picture.^46^ Early adversity can also accentuate responses to stress during later life, and this includes the risk for mental disorder and drug misuse.^47^, ^48^, ^49^, ^50^ Maltreated children show less trust, worse decision making, poorer preference choice, risk assessment and greater negativity bias to others as adolescents and adults than those with more uneventful backgrounds.^51^, ^52^ These long‐term consequences include changes in emotionality and cognitive style, personality and temperament and an increased risk for psychopathology.^33^, ^53^, ^54^, ^55^, ^56^, ^57^, ^58^, ^59^

Can we define more precisely the nature of this early sensitivity to adversity? The state of the brain and its reactivity during early life reflects the high number of genes expressed and the influence of the environment on them.^36^, ^60^, ^61^, ^62^ The endocrine environment, including adrenal steroids, has powerful effects on the development of the brain.^56^, ^63^, ^64^ The adrenal gland has a distinct post‐natal developmental trajectory in humans. During pre‐adrenarche, cortisol is secreted in amounts similar to adults. At adrenarche, this is joined by dehydroepiandrosterone (DHEA). The evidence presented in this paper suggests that this transition has highly significant effects on the way the young brain responds to adversity. Early sensitivity to adversity is coincident with a pre‐adrenarche period characterised by the secretion of adrenal cortisol unaccompanied by DHEA(S). Conversely, the occurrence of adrenarche coincides with the reduction in this sensitivity and enhanced stability of neural and psychological function. Are these events linked?

## 2 ADRENARCHE

There are several episodes of distinct neuroendocrine events during the human lifespan. One of the first is the secretion, by the foetal testis, of testosterone during the first few months of intra‐uterine life.^65^, ^66^ This seems to have significant effects on future sexuality, including gender identity and sexual orientation, but the evidence is still incomplete.^67^ Puberty represents a second transitional neuroendocrine episode, with dramatic and well‐known results on both physical and psychological functioning. Menopause is a third such event, signalling the end of female reproductive ability. Adrenarche is a fourth example, though it has no established function.

**FIGURE 1:**
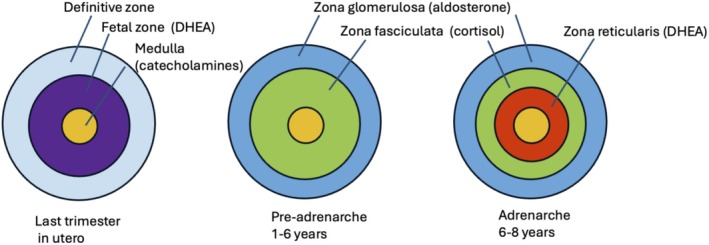
Diagrammatic representation of the changes in adrenal function during early life in humans.

Adrenarche is characterised by marked increases in blood levels of DHEA and its derivative DHEAS around 6–8 years of age, and has been repeatedly reviewed^68^, ^69^, ^70^, ^71^, ^72^, ^73^ (Figure 1). It is not the first exposure of the brain to DHEA(s) which is produced by the foetal zone of the adrenal but ceases at birth. Prenatal DHEA(S) supplies the placenta with the first steps in the synthesis of other steroids.^74^ The majority of papers on adrenarche are concerned with the phenomenon itself: what initiates it, its function—both still obscure—including the possibility it has behavioural or neural effects, and whether variation in the timing of adrenarche has any physiological or psychological significance.^68^, ^75^, ^76^ It is known to occur in humans as well as some apes, and anthropoid primates though generally not in rodents.^77^ The biological explanation for the occurrence of adrenarche in primates is an enigma. Possible explanations include the long duration of childhood, the development of larger brains, the increased ability for early learning and memory and moderation of the detrimental effects of exposure of the brain unrestricted glucocorticoids. Levels of other steroids, such as 11‐keto‐testosterone, increase during adrenarche, and may contribute to some of the androgenic phenomena such as the growth of pubic hair; there is no data on interactions with cortisol.^78^

Its immediate cause is activation of the adrenal zona reticularis, but how and why this occurs is still mysterious.^70^, ^79^ No hypothalamic or pituitary activators have been described, though the timing of adrenarche seems independent of puberty, indicating that they have separate mechanisms. No function has been established for adrenarche including any effects that increasing levels of DHEA(S) might have on brain function or possible interactions with the subsequent surge of gonadal hormones that characterize puberty.^80^ The endocrine profiles of adrenarche and puberty are quite different. Whereas puberty is defined by activation of the gonads, with the consequent increase in gonadal hormones such as testosterone, estrogen and progesterone, adrenarche is marked by the secretion of DHEA(S) from the adrenal.^79^, ^81^ Mean blood levels of cortisol continue largely unchanged from birth.^82^

## 3 PRE‐ADRENARCHE AND CORTISOL

The high levels of plasticity in the young brain account for much of the effects of early adversity upon it. Consequences include aversive memories of abuse or neglect and the effect these and similar contemporaneous events have on the formation of personality and temperament.^56^, ^83^ Experimental studies have shown that early disruption of maternal care can result in enhanced retention of fear‐related memories but impaired recognition and social memory, though findings have not always been consistent.^84^, ^85^

The pre‐adrenarche period is characterised by secretion of cortisol from the adrenal without accompanying DHEA(S). This is a unique circumstance in the human life‐span. DHEA(S) moderates many of the actions of glucocorticoids (see below). Contemporary levels of cortisol will therefore have more exaggerated actions than after adrenarche, and increased levels following stressful events will have greater impact than they would have in the presence of physiological levels of DHEA(S).^86^ The brain expresses both mineralo‐ and gluco‐corticoid receptors (MR, GR), the former being more restricted than the latter.^37^, ^87^ Corticoids also act on membrane‐bound receptors, though their function is still uncertain.^88^, ^89^ Cortisol has greater affinity for MR than GR, so their combined activity depends on the relative occupation or activation of these two receptors.^89^

There are marked changes in the patterns of gene expression in the human brain during the first few years of life.^62^, ^90^, ^91^, ^92^ This will influence the effect that the endocrine environment has on brain function. Many of these genes regulate cell adhesion and dendritic growth including members of the protocadherin and IGF family. Many (more than 80) contain glucocorticoid response elements (GRE) and so a wide spectrum of brain activity will be sensitive to cortisol in the pre‐adrenarche period.^93^, ^94^ Stress or increased cortisol during this time have persistent effects on the expression of genes in the brain, including CRHR1, CRHR2, FKBP5, and SLC6A4 and a range of immediate‐early genes^95^ which would alter psychological and physiological responses to subsequent challenges or loss.^29^, ^54^, ^96^, ^97^ Other corticoid‐sensitive genes include interleukin‐1β, serotonin (see below) and corticotrophin‐releasing factor.

Glucocorticoids have complex actions on memory. These include episodic memories that can recall instances of abuse as well as implicit memories for emotional events that may result in psychological consequences without conscious recall.^98^, ^99^, ^100^ They have marked effects on consolidation and retrieval of emotionally arousing experiences and fearful memories, but not neutral ones^101^, ^102^ in both humans and experimental animals.^103^ This has been associated with changes in dendritic and synaptic development and altered levels of BDNF and NGF in the hippocampus, amygdala, and related regions of the brain as well as glutamate/GABA balance.^104^, ^105^, ^106^ Some of these actions require the synergistic release of noradrenaline.^107^ Many of these studies have been on adults, and it is not always clear how far they apply to early life.^108^ Memories of early adversity have obvious relevance to a child's experience of early maltreatment and its influence on later mental health.^101^, ^109^, ^110^, ^111^

Glucocorticoids facilitate reorganisation of synapses by experiential events^112^, ^113^, ^114^, ^115^ and have marked effects on the function of BDNF, a major regulator of plasticity, and whose levels are highly dynamic in response to stress.^116^, ^117^, ^118^ This will have substantial effects on cognition and affective behaviours.^119^, ^120^ The trophic actions of glucocorticoids on the developing brain are largely a consequence of activating BDNF and tropomyosin‐related kinase B (TrkB), the principal BDNF receptor.^121^, ^122^ Experimentally, the expression of TrkB is necessary in the amygdala, hippocampus and frontal cortex for the formation of emotional memories.^123^ Glucocorticoids thus contribute to the encoding and persistence of engrams that underly adversity‐dependent patterns of behaviour or adaptive traits in personality or temperament.^124^, ^125^

The hippocampus plays a central role in the process of establishing memories and their recall.^126^, ^127^, ^128^ It also expresses high concentrations of both MR and GR.^129^, ^130^, ^131^ Corticoids have powerful effects on its function, including the formation and retention of stressful or emotional memories.^132^, ^133^, ^134^, ^135^ Adrenalectomy reduces dendritic formation in the hippocampus, which is restored by glucocorticoid treatment.^136^, ^137^ It is one of the few areas of the brain in which neurogenesis continues into adult life.^138^, ^139^ Debates continue about the functional implications of adult neurogenesis, though a role in the formation of new memories and forgetting juvenile ones is a credible one.^78^, ^140^, ^141^, ^142^ Other areas of the brain, including the amygdala and orbital frontal cortex, are also involved in the retention and consolidation of such memories, as well as the influence that early adversity has on the development of temperament and personality, and these are also sensitive to glucocorticoids.^143^, ^144^, ^145^, ^146^

Early life deprivation or abuse therefore results in long‐term changes in the structure and connectivity of the brain including epigenetic‐dependent alterations.^41^ There are individual differences in the sensitivity or resilience of individuals to early adversity, and these have been related to corresponding differences in methylation of corticoid receptor genes^29^, ^83^, ^147^ as well as other variants (see below). Similar variations have been associated with differences in emotion‐related and non‐declarative memory^148^, ^149^ and enhanced negative memories of adverse events.^150^, ^151^, ^152^ There will be long‐term effects on emotional reactivity and mood. These include linguistic and cognitive skills as well as decision‐making and risk‐taking in addition to an increased likelihood of impaired physical health, including altered immune function and cardiovascular disease.^153^ During the pre‐adrenarche period, there is no moderation of these actions of cortisol by DHEA(S).

## 4 MODERATION OF CORTICOIDS BY DHEA(S)

A prominent and powerful effect of DHEA(S), and one most relevant to the conjecture put forward in this paper, is its anti‐ glucocorticoid actions, which have been recognised for many years.^86^, ^154^, ^155^ These cover most of the known actions of glucocorticoids. They include the actions of glucocorticoids on adipose tissue,^156^, ^157^ immune function,^158^, ^159^, ^160^, ^161^ B and T lymphocytes,^162^, ^163^ cellular apoptosis,^164^ the suppression of IGF1,^165^ apoptosis and thymus function,^163^, ^164^ the response to stress,^166^, ^167^ the formation of memories,^168^ the action of corticoids on heart muscle,^169^ the neurotoxic actions of corticosterone in hippocampal cell cultures^170^, ^171^ and suppression of neurogenesis in the adult hippocampus.^172^ The mechanism whereby DHEA or its principal metabolites, which include 7α‐OH‐DHEA, 5‐androstenediol, and androstenedione, achieve this is not established.^173^ All these moderating actions will reduce the sensitivity of the young brain to cortisol and to adverse events. DHEA(S) also decreases the production of cortisol from its metabolites (e.g., cortisone) by 11β‐HSD1^174^ but efforts to show altered GR activation or trafficking or even a direct action on glucocorticoid receptors of DHEA(S) have been unsuccessful.^175^, ^176^ DHEA thus moderates the action of glucocorticoids on early development of the brain,^177^ reduces the effect that higher levels of pre‐natal maternal cortisol have on post‐natal negative emotional responses in babies^178^ and protects human subjects against stress‐induced changes in personality.^179^ The marked reduction in hippocampal neurogenesis by glucocorticoids^180^, ^181^, ^182^ is also prevented by the simultaneous administration of DHEA in rats, which ordinarily secrete little of it,^172^, ^183^ possibly via the Akt signalling pathway.^184^ The advent of DHEA(S) at adrenarche therefore attenuates the actions of cortisol across its entire spectrum in both basal and stress‐induced contexts. One exception may be negative feedback on ACTH levels from the pituitary, since cortisol levels do not change during adrenarche.

There is no other epoch of the human life span in which cortisol acts without significant amounts of DHEA(S). During this pre‐adrenarche period, cortisol will have a unique access to brain mechanisms responsible for memory, cognition, personality, emotionality, all consequent upon associated neural mechanisms including synaptic modifications and plasticity but unaffected by DHEA(S). This will have marked effects on the sensitivity of the brain to environmental events, including adversity. This would also apply to stress‐induced increases in cortisol (e.g., in response to abuse, etc),^185^ which would have an amplified action on the brain in the absence of moderation by DHEA(S).

## 5 ADRENARCHE AND DHEA

A number of features distinguish DHEA from other steroids, including cortisol and those from the gonads. DHEA in the blood has two forms: free DHEA and a sulphated derivative (DHEAS). Whereas levels of DHEA in the adult fall within the range of other steroids, those of DHEAS are orders of magnitude higher.^186^ Free DHEA enters the brain, like other steroids, but DHEAS is largely excluded.^187^, ^188^, ^189^

Unlike cortisol, there are four epochs in the life‐time trajectory of DHEA. It is secreted in large amounts from the foetal adrenal,^86^ but this drops to almost zero at birth.^190^ As pointed out above, it is important to note that the post‐natal pre‐adrenarche interval is the only epoch in the human life cycle when any moderating effect of DHEA on cortisol is mostly absent. Adrenarche is represented by a surge of DHEA(S) at around 6–8 years.^69^, ^70^, ^191^ By the early 20s, DHEA(S) levels are at their highest, only to decline progressively (but variably), so that by age c. 60 years they are around 30% of what they were in young adults.^192^, ^193^, ^194^ One consequence is that relative levels of cortisol and DHEA (S) vary markedly across each of these epochs. The effect these will have on brain function depends not only on contemporary cortisol and DHEA(S) levels (their molar ratio) but also on whatever actions there might have been during previous epochs.^195^, ^196^ There are reports that DHEA(S) and associated steroids (e.g., pregnenolone) may be produced within the brain by both glial cells and neurons^197^, ^198^, ^199^, ^200^ though this is still controversial, and there is no information about how this might change during the life span; for example, at adrenarche. The presence of CYP17A1 in human frontal lobes may indicate DHEA synthesis, but this enzyme is also part of the precursor pathway for corticoids and testosterone.^201^

There is no evidence that DHEA or its sulphated derivative, unlike cortisol, binds to a specific intracellular receptor. Numerous reports show DHEA interacting with TrkA, NGF, GABA‐A, NMDA, sigma, pregnane X and a variety of G‐coupled receptors, as well as those binding oestrogens and androgens.^164^, ^202^, ^203^, ^204^, ^205^, ^206^ It also increases BDNF concentrations and binds to TrkB receptors^207^, ^208^ which suggests a positive action on neural function.^209^ It moderates both serotonin and dopamine activity.^210^ DHEAS can act as a GABA‐A channel‐blocker.^211^ This spectrum discourages assigning any action of DHEA(S) on the brain to a particular cellular mechanism but illustrates its potent action on brain plasticity and development. DHEA is an inhibitor of glucose‐6‐phosphate dehydrogenase (G6PDH), an essential step in the pentose shunt (phosphate) pathway that produces NADPH.^212^ This has a significant role in protection against oxidative stress and the production of nucleotides.^213^, ^214^ Reduced oxygen‐free radicals would also reduce inflammation.^215^ Whether this plays a role in brain function, particularly the production of cytokines and their putative role in neural plasticity in the post‐adrenarche period remains to be determined (see below).

A correspondingly wide range of functions has been attributed to DHEA(S), including neuroprotective, antioxidant, antihypertensive, and anti‐inflammatory properties as well as postulated anti‐depressive and anxiolytic actions and positive effects on a variety of cognitive and emotional abilities, including memory.^216^, ^217^, ^218^, ^219^, ^220^ In rodents, administration of DHEA counteracts some of the deleterious effects of earlier corticoids on neuropsychological function.^221^ DHEA reduces the activity of the amygdala, hippocampus, and anterior cingulate cortex and memories for emotional stimuli.^219^ These actions are the converse of those of cortisol.^222^, ^223^

The panoply of functions associated with DHEA(S), listed above, is entirely compatible with the possibility that adrenarche could also function to moderate the early sensitive period by actions that are, at least in part, independent of cortisol. These include those on BDNF, the immune system, perineuronal nets (see below) and the many genes that are highly active in the early brain, some not regulated by cortisol. There are also well‐established actions of DHEA(S) on mood and emotional states, which may be relevant to psychological reactions to adversity or stress.^179^, ^224^

The action of cortisol during the pre‐adrenarche period undoubtedly involves synaptic re‐organisation and new connections. This represents the effects early adversity has on memory and on emotionality and personality. The addition of DHEA not only opposes these actions but also has a positive effect on consolidation of previous changes in brain structure that have resulted from early adverse events by another set of actions on synapses and connectivity.^225^ Whether adult hippocampal neurogenesis plays a role in the impact of early adversity on brain function is not known (see above). Consolidation of beneficial events in early life is an advantageous action of DHEA, but similar actions on adverse ones is a detrimental side‐effect.

## 6 ADRENARCHE AND GABA

Shaping patterns of connectivity is influenced both by the physiological environment (including hormones and growth factors), by genetic factors, and by different types of experience.^226^ The onset and ending of a sensory sensitivity depend on changes in inhibitory mechanisms, in which GABA plays a central (but not exclusive) role.^227^ GABA depolarises neurons in immature brains, rather than hyperpolarises them as in adults.^228^ One result is the co‐activation of NMDA glutamate receptors. This may encourage neuronal assembly and synaptogenesis. The development of GABAergic parvalbumin‐expressing interneurons indicates the switch between excitatory and inhibitory functions of GABA and the closure of critical periods.^229^ The onset and termination of the critical period in the visual system is altered by manipulating GABA activity.^230^, ^231^, ^232^

The genes (Gad65, Gad67) synthesising GABA (via glutamic acid decarboxylase) contain a glucocorticoid response element.^233^, ^234^ Ocular dominance plasticity is prevented in mice by knocking these genes out but accelerated by GABA receptor activation (by benzodiazepines) as well as by over‐expression of BDNF.^235^ Glucocorticoids are potent regulators of GABA.^236^, ^237^ Chronic stress, with its accompanying elevation of glucocorticoids, decreases levels of glutamic acid decarboxylase and GABA function.^238^, ^239^ The pre‐adrenarche period is therefore characterised by relative lack of inhibitory GABAergic activity, and an enhancement of neuroplasticity. DHEA is a negative allosteric modulator of the GABA‐A receptor and therefore enhances inhibitory neural mechanisms.^236^, ^240^ GABAergic parvalbumin expressing interneurons, however, are also characterised by forming perineuronal nets.

## 7 STEROIDS AND PERINEURONAL NETS

Perineuronal nets are a prominent extracellular feature of the brain. They are formed from the brain's extracellular matrix (ECM) and are composed largely of chondroitin sulphate proteoglycans.^241^ They preferentially (but not exclusively) surround the soma, axonal initial segment, and proximal dendrites of parvalbumin‐expressing (PV+) interneurons and have been detected in many regions of the brain.^242^, ^243^ In humans they begin to be formed around 2 years but only achieve adult levels at around 8 years.^244^ This is the time that sensitivity to early adversity decreases. It also coincides with the advent of adrenarche. Are these three events linked?

One of the functions of perineuronal nets is to stabilise synapses and limit the formation of new ones.^245^ They also encourage the formation of erasure‐resistant fear‐related memories and thus close a postnatal, pre‐adrenarche period during which such memories can be erased.^246^ They play a central role in plasticity, and this may underlie their many actions, including those on memory, drug addiction and neurodegeneration.^241^, ^247^, ^248^ There is already evidence that perineuronal nets are a significant regulator of critical periods.^249^, ^250^ Monocular deprivation in young animals leads to an ocular dominance shift, but not in adults.^251^ However, removal of perineuronal nets by the enzyme chondroitinase restores this critical period, and preventing their formation by knocking out Crl1, which triggers their development, prolongs the ocular critical period and resets the rodent visual system to a more immature state.^248^, ^250^, ^252^ Astrocytes play a major role in the maturation of the brain and the appearance of perineuronal nets.^253^ The appearance of perineuronal nets at adrenarche will, therefore, have at least two major actions: limit the sensitivity of the young brain to adverse events, but also consolidate such memories of those events that may have been formed during the pre‐adrenarche period.^254^ This may account for the increased difficulty of reversing longer‐term effects of early adversity in older children.^255^

Corticoids, such as methylprednisolone or dexamethasone, inhibit the formation of perineuronal nets. A spectrum of genes has been implicated in their formation. This includes Otx2 and Sema3A, as well as hyaluronan synthase and metallopeptidase 9 (MMP9).^256^, ^257^ All are reduced by glucocorticoids, and therefore the pre‐adrenarche period, in which cortisol is unopposed by DHEA, will actively discourage the appearance of perineuronal nets.^247^, ^258^, ^259^, ^260^ Furthermore, stress associated with abuse, with its accompanying increase in glucocorticoids, also reduces perineuronal nets.^261^, ^262^, ^263^, ^264^ MMP‐9, released by glia and neurons, is directly responsible for PNN degradation, as shown by increased densities of perineuronal nets following genetic deletion of the MMP‐9 gene in mice.^265^, ^266^ Hyaluronan is a prominent and essential component of perineuronal nets. Glucocorticoids suppress the activity of HAS3 (hyaluronan synthase) and this is one way that they discourage the formation of perineuronal nets.^251^, ^267^, ^268^ CX3CR1 is another essential element, and it is also suppressed by glucocorticoids.^269^ The formation of perineuronal nets is therefore inhibited during the pre‐adrenarche, cortisol‐dominated period.

The role of perineuronal nets in fear‐related memory is particularly relevant to the long‐lasting effects of early adversity. These memories have been associated with activity in the basolateral amygdala during an early critical period and removal of perineuronal nets in this part of the brain enables the early phase to be reinstated and such memories to be erased.^246^, ^270^, ^271^ It also increases generalisation of aversive memories, a hallmark of some psychiatric disorders, by an action on parvalbumin‐expressing neurons (associated with perineuronal nets) in the amygdala.^272^ The establishment of perineuronal nets at the end of the critical period may be instrumental in stabilising synapses and preserving malignant (as well as beneficial) memories. In this way, adrenarche may consolidate such memories, set up by earlier maltreatment, with consequences for later mental health. Early adversity or stress (for example, consequent upon abuse) may also alter the formation of perineuronal nets in the adult rodent with additional effects on longer‐term memories.^262^, ^273^, ^274^

Are any of these actions of glucocorticoids moderated by DHEA(S)? No attention has been given to the possibility that adrenarche might influence the formation of perineuronal nets, so no attempt has been made to determine experimentally whether DHEA(S) or related steroids (e.g., pregnenolone) might moderate the actions of glucocorticoids on perineuronal nets. Given the wide range of opposing actions that DHEA(S) has on corticoids, it seems a reasonable a priori assumption. Suggestive evidence points to GABA‐A receptors (see above). GABA has been implicated in the onset and termination of critical periods observed both in the visual system and more generally.^10^, ^231^, ^275^ GABA‐A receptors are closely associated with parvalbumin‐expressing neurons and perineuronal nets and there is considerable evidence that DHEA(S) has powerful actions on these receptors.^276^, ^277^, ^278^ DHEAS binds to the GABA‐A receptor and is a negative allosteric modulator.^211^ The expression of BDNF is decreased by corticoids, and by persistent stress, in some areas of the brain^279^ but increased by DHEA and this would be consistent with the formation of perineuronal nets with consequent reduction in sensitivity to external events and enhanced stability of synapses.^116^, ^201^, ^220^, ^280^ One caveat is that laboratory rodents do not secrete appreciable amounts of DHEA, so experimental attempts to test the effects of DHEA(S) on perineuronal nets on them would be pharmacological rather than physiological. It is obvious that while glucocorticoids have an established role in their formation, more investigation of the effects of adrenarche and DHEA(S) on the generation and function of perineuronal nets is needed.

## 8 THE ROLE OF SEROTONIN

Serotonin is widespread in the brain, with a correspondingly wide spectrum of neural activity. Here the focus is on its function as a moderator of sensitivity to early adversity, and whether this is altered by adrenarche. Serotonin levels increase during the first 2 years, but decline after around 5 years.^281^ They are a crucial element in the cascade of events that underlie early brain development.^282^ There is a considerable literature showing that serotonin diminishes sensitivity to aversive sensory events, regulates plasticity of the brain and memory function in concert with glucocorticoids^282^, ^283^, ^284^ as well as having a role in the emotional responses to them and susceptibility to longer‐term consequences.^285^, ^286^, ^287^, ^288^ Serotonin has an established role in fear conditioning, highly relevant to the effects of early maltreatment, and this is also sensitive to glucocorticoids.^289^, ^290^, ^291^

Much attention has been on the serotonin transporter (SERT, 5‐HTT, SLC6A4), which regulates the reuptake of serotonin following its release into the synaptic cleft. A common genetic variation in SERT is a repeat length polymorphism in the promoter region (5HTTLPR) which has been associated with increased vulnerability to, and coping with, early life psychosocial stress in rodents and humans,^203^, ^292^, ^293^, ^294^, ^295^ to the risk of experiencing emotional difficulties following early life deprivation^296^ and for depression in adolescents.^26^ Persistent stress upregulates SERT in the brain via increased glucocorticoid levels.^297^ Epigenetic methylation of SERT is enhanced by early stress, but this is modulated by its genotype.^298^

There are seven families of serotonin receptors, but 5HT1A seems particularly sensitive to longer‐term actions of glucocorticoids.^27^, ^299^ The 5HT1A gene has a glucocorticoid response element which represses its expression,^300^, ^301^ which would be the case in the pre‐adrenarche period. Any interpretation of what this might mean for neural function is complicated by 5HT1A being both pre‐synaptic (e.g., raphe) and post‐synaptic (e.g., hippocampus), often with opposing actions on neural activity. Post‐synaptic 5HT1A receptors are reduced by stress, but this is counteracted by adrenalectomy, suggesting tonic inhibition by adrenal steroids.^167^ A further consideration is that glucocorticoids can also regulate other 5HT receptors, including 5HT2C.^302^ It is evident that genetic variations in SERT together with corticoid‐influenced actions on 5HT receptors have marked consequences for the impact of early adversities on the brain during the pre‐adrenarche period.

The scanty literature on the actions of DHEA on serotonin is consistent with it having excitatory or synergistic actions on serotonin, but more detailed studies are needed. Higher levels of endogenous DHEA (S) levels potentiate the anti‐depressive actions of serotonin‐acting SSRIs.^303^ DHEA also antagonises the inhibitory action of GABA on dorsal raphe (serotonin) neurons.^277^ DHEA administered to rats increased the turnover of serotonin in the striatum^304^ and reduced the activity of monoamine oxidase, a principal agent in the breakdown of amines including serotonin.^305^ What evidence there is points to adrenarche increasing serotonin activity and thus decreasing the impact of sensory events.

## 9 STEROIDS AND CYTOKINES

Corticoids are generally considered to repress systemic inflammation and are widely used clinically for such a purpose. However, they appear to be pro‐inflammatory in the brain.^306^, ^307^, ^308^, ^309^, ^310^ Inflammation is a term usually associated with pathology. Production of cytokines is part of this process, but it is becoming apparent that they, and the cells that produce them (e.g., microglia) have a physiological function in the brain, particularly on synaptic remodelling and plasticity.^311^ This includes the tumour necrosis factor family, interferons, interleukins, and chemokines.^312^ Cytokines have powerful effects on neural function, but their actions are highly complex. There is a large number of cytokines, each with distinct and sometimes opposing actions on synaptic function and neural plasticity.^313^ A principal concept that has evolved in the field is that IL‐1β is the final common effector for many cytokine networks modulating memory. It is commonly believed that inflammatory cytokines facilitate or impair neuronal functions at physiologically low and pathologically high concentrations, respectively.^314^

It might be expected that the unopposed actions of cortisol during pre‐adrenarche would be reflected in higher levels of either blood or brain cytokines, but there is no evidence for this.^315^ DHEA has anti‐inflammatory actions and thus moderates that of cortisol at adrenarche including reducing microglial activation.^202^, ^316^ DHEA reduces microglial inflammatory responses,^317^, ^318^ opposing the actions of corticoids on immune function^319^, ^320^ and hence cytokine production in the brain possibly via Akt 1 and 2 mediation.^202^ However, children exposed to early‐life adversities had higher TNF‐α and IL1β than controls. They also showed both raised DHEA and cortisol.^17^ Cytokines act on both NMDA and GABA receptors, and in this way influence synaptic pruning and Hebbian plasticity. Deficient microglia activity impairs neural connectivity, memory and subsequent social interactions.^313^, ^314^, ^321^, ^322^ These are some of the later hallmarks of early adversity. The advent of adrenarche therefore diminishes the effects of glucocorticoids on synaptic function.

## 10 ADRENARCHE AND SENSITIVITY TO ADVERSITY

Pre‐adrenarche is a period of intense and rapid development of the brain.^60^, ^61^, ^323^, ^324^ This includes the enhanced ability for learning and adaptation. Underlying this is increased sensitivity and reaction to potentially damaging environmental events. Cortisol is one contributor to this neural state but is not the only one. Many genetic and associated processes occur during this time, only some of which are influenced by cortisol. Nevertheless, an epoch during which cortisol is not moderated by DHEA(S) would add significantly to the plasticity and reactivity of the young brain. Other elements collaborate in this process, including reduced serotonin activity, the relative absence of perineuronal nets, activation of microglia and the consequent release of cytokines and related molecules; all will add further reactive capacities to the early brain. Cortisol has a significant but not exclusive influence on these processes.

At adrenarche many of these mechanisms would be reduced or inhibited. All the available evidence suggests that DHEA(S) has many powerful attenuating actions on cortisol, as well as direct actions on the brain which counter those of cortisol. The overall action of DHEA(S) would be to reduce the effects that adverse or malign events have on the developing brain. This is not to say that adversity has no psychological or neural consequences after adrenarche, but the evidence is clear that such events during early life represent an enhanced risk for later neural malfunction and psychological vulnerability to later adversity or challenges. It has to be recognised that much of the evidence supporting the ideas presented here is based on data from experiments on animals, extrapolated to humans, though it should also be noted that much fundamental neuroendocrinology that does apply to humans was discovered in the laboratory.

The remarkable ability of the young child to learn new cognitive, social and motor skills has already been mentioned.^325^, ^326^ It is entirely possible that this might also be influenced by adrenarche. A period of relative stability, following the essential and rapid adaptation to the infant's new world would be advantageous. There is no doubt that rapid and accurate acquisition of these skills is an essential part of successful rearing. Accentuated sensitivity to adverse events is thus a downside of an important early neural ability that may also be limited by adrenarche. However, an adequate consideration of this aspect of adrenarche lies outside the scope of this paper. It should be noted that, in contrast to the notion presented here, DHEA(S) has been suggested to prolong neural plasticity during pre‐adolescence.^73^ However, plasticity is greatest during the immediate postnatal period, and the final stages of synaptic development occur in the early 20s, when DHEA(S) is at its highest levels.^231^, ^327^, ^328^

There has been little focus on the role of adrenarche in early neural sensitivity, and much further evidence needs to be obtained. The conjecture offered here is that the sequence of pre‐and post‐adrenarche has far‐reaching consequences for responses to environmental events during early life and therefore for later neural and mental function and the way the brain responds to additional challenges. It further suggests that the transition from pre‐adrenarche to adrenarche is a developmental critical neuroendocrine event that is as significant for neural function as the onset of puberty.

## CONFLICT OF INTEREST STATEMENT

The author declares no conflicts of interest.

## Data Availability

Data sharing not applicable to this article as no datasets were generated or analysed during the current study.
